# Does the use of N-butyl-2 cyanoacrylate in the treatment of lower extremity superficial varicose veins cause acute systemic inflammation and allergic reactions?

**DOI:** 10.5830/CVJA-2018-012

**Published:** 2018

**Authors:** Korkmaz Özge, Göksel Sabahattin, Berkan Öcal, Gül Müslim, Başçil Hasan, Yildir Yavuz

**Affiliations:** Department of Cardiovascular Surgery, Cumhuriyet University School of Medicine, Sivas, Turkey; Department of Cardiovascular Surgery, Cumhuriyet University School of Medicine, Sivas, Turkey; Department of Cardiovascular Surgery, Cumhuriyet University School of Medicine, Sivas, Turkey; Sivas Numune State Hospital, Sivas, Turkey; Sivas Numune State Hospital, Sivas, Turkey; Department of Medical Biology, Cumhuriyet University, Sivas, Turkey

**Keywords:** N-butyl-2 cyanoacrylate, lower-extremity superficial varicous vein, acute systemic inflammation, allergic reaction

## Abstract

**Introduction:**

In this study we used N-butyl-2 cyanoacrylate (NBCA), including dimethyl sulfoxide (DMSO), via the endovenous route, for mechanochemical ablation in the treatment of superficial venous insufficiency, in an attempt to establish whether an early systemic inflammatory response and an allergic reaction occurred in the patients.

**Methods:**

A total of 102 patients were treated with endovenous medical ablation in two centres between October 2015 and February 2016. This study was a two-centre, retrospective, non-randomised investigational study. Ablation treatment with endovenous NBCA was used in patients with C3 to C4b grade superficial venous insufficiency, according to the CEAP (clinical, aetiology, anatomy and pathophysiology) clinical classification, with sapheno-femoral junctional insufficiency and a reflux of 0.5 seconds and longer on duplex ultrasonography. Pre-operative whole blood count, erythrocyte sedimentation rate (ESR), C-reactive protein (CRP) level and blood chemistry were studied in all patients on admission to the clinic, and repeated in the second hour post-intervention.

**Results:**

All patients were treated successfully. Pre-operative white blood cell count (WBC) was 6.82 ± 1.67 × 109 cells/μl, and post intervention it was 6.57 ± 1.49 × 109 cells/μl; the difference was not statistically significant (p = 0.68). The neutrophil count before the intervention was 4.09 ± 1.33 × 109 cells/μl, while afterwards, it was 4.09 ± 1.33 × 109 cells/μl, with no statistically significant difference (p = 0.833). Pre-intervention eosinophil count was 0.64 ± 1.51 × 109 cells/μl, while it was 0.76 ± 1.65 × 109 cells/μl after the intervention, and the difference was statistically significant. Pre-intervention ESR and CRP values were 18.92 ± 9.77 mm/h and 1.71 ± 1.54 mg/dl, respectively. Postoperative ESR and CRP values were 19.78 ± 15.90 mm/h and 1.73 ± 1.59 mg/dl, respectively, but the differences were not statistically significant. When the parameters were analysed by gender, the differences between pre- and postoperative WBC and eosinophil count, ESR and CRP in women were not statistically significant. On the other hand, although the change in WBC count and CRP value were not statistically significant in males, the differences in eosinophil count and ESR were statistically significant.

**Conclusion:**

Cyanoacrylate has been used in the endovenous medical ablation of varicose veins and superficial venous insufficiency over the last few years without the use of thermal energy and tumescent anaesthesia, which represents the greatest advantage of this method. In addition, since it causes no systemic allergic or acute inflammatory reaction, it appears to be safe to use.

Lower extremity venous insufficiency and the secondary development of varicose veins are important health problems that are frequently encountered in society. They impair the quality of life of individuals, and in certain conditions cause severe complications. The prevalence of venous insufficiency has been reported to be between 20 and 40% in many studies.^1^,^2^

Surgery has been the preferred method of treatment for this disease for more than 100 years. However, due to postoperative complications and frequent recurrence, alternative methods of treatment have been sought. Newly developed endovascular techniques have gradually replaced open surgery during the last two decades.

Haematoma, paresthesia, wound site scars, deformities, and a high rate of recurrence are among the complications of surgery.^3^,^4^ Minimally invasive endovenous thermo-ablation techniques (radiofrequency and laser), applied in the last decade in the treatment of superficial venous insufficiency and varicose veins, have decreased postoperative complications, shortened the healing process and improved quality of life.^5^ However, the necessity of tumescent anaesthesia during these techniques, and complications in the postoperative period, such as pain, ecchymosis and paresthesia caused by perforation of the vein wall, have limited the use of these techniques.^6^,^7^

The introduction of cyanoacrylate (CA) in medical applications dates back to the 1960s. Surgeons used CA in order to stop bleeding and close wounds during the Vietnam War.^8^ Also, endoscopic CA injection to stop gastric variceal bleeding has been safely and widely used.^9^ Recently, it has been used in the closure of type I and II endoleaks developing during the repair of abdominal aortic aneurysms, varicoceles, pelvic congestion syndromes and arteriovenous malformations.^10^

N-butyl-2 cyanoacrylate (NBCA) has been used via the endovenous route in the treatment of venous insufficiency and varicose veins, with the aim of biochemical ablation.^11^ NBCA rapidly hardens in a polymerisation reaction following intravenous injection and occludes the vein. In addition, it causes a local inflammatory reaction in the vein wall and surrounding tissues.^10^,^12^ However, there are no studies in the literature evaluating whether NBCA causes systemic inflammation following contact with the blood circulation.

We attempted to establish whether NBCA caused a simultaneous systemic inflammatory response in the early period while causing a local inflammatory reaction in the vein wall and surrounding tissues in patients who were administered NBCA, including dimethyl sulfoxide (DMSO), for the treatment of superficial venous insufficiency. We retrospectively evaluated preand post-interventional blood samples in order to determine this.

## Methods

This study was a two-centre, retrospective, non-randomised investigational study. Ablation treatment with endovenous NBCA was applied to patients with C3 to C4b grade venous insufficiency, according to the CEAP (clinical, aetiology, anatomy and pathophysiology) clinical classification, with saphenofemoral junctional insufficiency and a reflux of 0.5 seconds and longer on duplex ultrasonography, between October 2015 and February 2016. This treatment was abandoned in patients with a greater saphenous vein diameter of > 15 mm and < 5 mm.

The treatment is contra-indicated in patients who have a past history of deep venous thrombosis, have femoral vein insufficiency, congenital vasculopathy, thrombophilia, the presence of severe systemic disease, and in pregnant and lactating patients. This procedure was not used in any patient who had any of these conditions.

Detailed demographic data of the patients who were treated using endovenous NBCA ablation therapy were collected. Whole blood count, sedimentation rate, C-reactive protein (CRP) and blood chemistry were studied in all patients on admission to the clinic. These examinations were repeated in the second hour post-intervention.

Patients who were taken into the operating room to undergo endovascular medical ablation were monitored by the anaesthesiology team. Subsequently, both legs were re-evaluated with Doppler ultrasonography. The integrity of the iliac vein and inferior vena cava in the abdominal region was confirmed in order not to overlook some rare conditions, such as possible inferior vena cava agenesis.

Patients were placed in the supine position and the leg and inguinal region were cleaned and draped in order to perform the intervention under sterile conditions. With the aid of Doppler ultrasonography, an appropriate segment of the greater saphenous vein was selected for catheterisation, and a 5F introducer sheath was placed following local anaesthesia. The placement of the catheter was confirmed by ultrasonography.

A 0.035-inch J guidewire was advanced into the sheath. Ultrasonography was used to determine whether the guidewire had reached the sapheno-femoral junction and a 4F carrier catheter was advanced into it. The catheter was confirmed to be at the sapheno-femoral junction and then withdrawn 3 mm, and a 3-ml syringe and piston system, which provides NBCA injection, was positioned. The location of the catheter was checked again by ultrasonography and it was confirmed not to be in the sapheno-femoral junction. The junction was then compressed by the ultrasonography probe and obstructed.

The piston of the syringe administered 0.3 ml of NBCA during each pulse into the saphenous vein and compression was performed simultaneously. Intravenous administration of NCBA, which provided medical ablation, was continued while the catheter was withdrawn rapidly at a rate of 2 cm/s. At the end of the procedure, compression was continued for five to 10 seconds and the procedure was terminated after the saphenofemoral junction was demonstrated by ultrasonography to be open and the rest of the greater saphenous vein was occluded.

A compression sock was placed on the leg that underwent the procedure and medium pressure was applied. The patient was taken to the ward for follow up and repeat testing of the whole blood count, blood chemistry, CRP and sedimentation rate. Patients with no complications in the eighth hour postoperatively were discharged with a follow-up plan of visits on the 10th day, and one, three, six and 12 months postoperatively.

## Statistical analysis

The results were evaluated using SPSS version 17. Changes in the patients’ values were calculated using the paired-samples t-test. The α-value was accepted as 0.05. The change in values by gender was calculated using the independent-samples t-test (p < α was accepted as significant).

## Results

A total of 102 patients were treated with endovenous medical ablation at two centres between October 2015 and February 2016. The mean age of the patients was 51.16 ± 1.17 years (range: 25–74); 72 (70.6%) were female and 30 (29.4%) were male. The mean diameter of the saphenous vein was 7.72 ± 2.02 mm (range: 6–14). Among the general risk factors, a positive family history was present in 31 cases (30.3%), use of tobacco products in 17 (16.7%), hypertension in six (5.9%), abnormal lipid profile in 19 (18.7%), obesity in 24 (23.5%) and diabetes mellitus in five cases (4.9%) (Table 1).

**Table 1:** Demographics of the patients

*Demographic data*	*Number (%)*
Age (years)	51.16 ± 1.17
Gender (female/male)	72 (70.6)/30 (29.4)
Presence of family history	31 (30.3)
Use of tobacco products	17 (16.7)
Hypertension	6 (5.9)
Abnormal lipid profile	19 (18.7)
Obesity (BMI ≥ 30 kg/m2)	24 (23.5)
Diabetes mellitus	5 (4.9)
CEAP classification	
C3	42 (41.2)
C4a	37 (36.3)
C4b	23 (22.5)
Vein diameter (mm)	7.72 ± 2.02

BMI: body mass index; CEAP: clinical, aetiology, anatomy and pathophysiology.

When the distribution of CEAP classification was analysed, 42 (41.2%) patients were found to be C3 grade, 37 (36.3%) were C4a, and 23 (22.5%) were C4b. All patients were treated successfully. Pre-operative white blood cell count (WBC) was 6.82 ± 1.67× 109 cells/μl, while after the intervention it was 6.57 ± 1.49 × 109 cells/μl; the difference was not statistically significant (p = 0.68). The neutrophil count before the intervention was 4.09 ± 1.33 × 109 cells/μl, and post intervention it was 4.09 ± 1.33× 109 cells/μl, with no statistically significant difference (p = 0.833). The eosinophil count was 0.64 ± 1.51 × 109 cells/μl prior to the procedure, and after the procedure it was 0.76 ± 1.65 × 109 cells/ μl. The difference was statistically significant, demonstrating a negative correlation (p = 0.01) (Table 2).

**Table 2:** Pre- and postoperative changes in whole blood and serological parameters of patients

*Parameter*	*Pre-operative Value*	*Postoperative Value*	*Amount of Change*	*t-value*	*p-value*
WBC (× 103 cells/μl)	6.82 ± 1.67	6.57 ± 1.49	0.24 ± 1.33	1.846	0.068
Neutrophils (× 103 cells/μl)	4.09 ± 1.33	4.09 ± 1.33	–0.00 ± 0.02	0.211	0.833
Eosinophils (× 103 cells/μl)	0.64 ± 1.51	0.76 ± 1.65	–0.11 ± 0.46	–2.624	0.010*
Basophils (× 103 cells/μl)	0.14 ± 0.43	0.06 ± 0.13	0.08 ± 0.42	1.966	0.052
Haemoglobin (g/dl)	13.79 ± 1.47	13.84 ± 1.61	–0.05 ± 0.80	–0.503	0.617
Haematocrit (%)	42.00 ± 3.82	42.54 ± 7.11	–0.53 ± 5.78	–0.656	0.515
Platelets (× 103 cells/μl)	238.01 ± 64.33	225.40 ± 78.97	12.61 ± 75.39	1.285	0.204
Sedimentation (mm/h)	18.92 ± 9.77	19.78 ± 15.90	–0.86 ± 12.29	–0.709	0.480
CRP (mg/dl)	1.71 ± 1.54	1.73 ± 1.59	–0.21 ± 0.59	–0.634	0.714
Rheumatoid factor (IU/ml)	6.89 ± 5.52	6.65 ± 4.92	0.23 ± 3.80	0.634	0.527

WBC: white blood cells; CRP: C-reactive protein. *p < 0.05 statistically significant.

Pre-intervention sedimentation rate and CRP values were 18.92 ± 9.77 mm/h and 1.71 ± 1.54 mg/dl, respectively, and postoperative values were 19.78 ± 15.90 mm/h and 1.73 ± 1.59 mg/dl, respectively. The differences were not statistically significant (psedim = 0.480, pCRP = 0.714). The change in values preand post intervention are presented in detail in Table 2.

The change in values by gender are summarised in Table 3. Differences in pre- and postoperative WBC and eosinophil count, sedimentation rate and CRP were not statistically significant in women. On the other hand, although the change in WBC count and CRP value was not statistically significantly different in males, the difference in the eosinophil count was statistically significant, with a negative correlation (p = 0.002). The difference in sedimentation rate was also statistically significant and demonstrated a positive correlation (p = 0.005) (Table 3). In other words, postoperative sedimentation rate decreased in men and the change was 2.66 ± 4.76 mm/h, which was statistically significantly different (p < 0.05) (Table 3). When the difference in the rheumatoid factor was evaluated pre- and postintervention, no statistically significant changes were found in either gender (p < 0.05) (Table 3).

**Table 3:** Pre- and postoperative changes in whole blood and serological parameters by gender Parameter Pre-operative value Postoperative

*Parameter*	*Pre-operative Value*	*Postoperative Value*	*t-value*	*t-value p-value*
WBC (× 103 cells/μl)				
Female	6.87 ± 1.55	6.65 ± 1.53	1.375	0.173
Male	6.68 ± 1.96	6.37 ± 1.41	1.262	0.217
Neutrophils (× 103 cells/μl)				
Female	3.99 ± 1.30	3.99 ± 1.30	–0.985	0.328
Male	4.35 ± 1.39	4.34 ± 1.38	1.116	0.273
Eosinophils (× 103 cells/μl)				
Female	0.51 ± 1.03	0.51 ± 0.98	0.000	
Male	0.96 ± 2.28	1.37 ± 2.57	–3.479	1.000
Basophils (× 103 cells/μl)				
Female	0.06 ± 0.16	0.04 ± 0.07	1.199	0.235
Male	0.33 ± 0.74	0.09 ± 0.22	1.740	0.093
Haemoglobin (g/dl)				
Female	13.17 ± 0.93	13.05 ± 0.89	1.000	0.324
Male	15.26 ± 1.51	15.76 ± 1.32	–2.584	0.022*
Haematocrit (%)				
Female	40.64 ± 2.77	39.78 ± 2.76	1.837	0.075
Male	45.29 ± 4.08	49.16 ± 9.74	–1.650	0.121
Thrombocytes (103 cells/μl)				
Male	186.11 ± 33.16	193.72 ± 38.98	–2.061	0.055
Sedimentation rate (mm/h)				
Female	22.08 ± 9.42	24.41 ± 16.79	–1.407	0.164
Male	11.33 ± 5.54	8.66 ± 3.07	3.065	0.005*
CRP (mg/dl)				
Female	1.24 ± 1.25	1.30 ± 1.26	–1.540	0.128
Male	2.84 ± 1.62	2.75 ± 1.85	0.526	0.603
Rheumatoid factor (IU/ml)				
Female	6.80 ± 5.83	6.41 ± 5.63	0.853	0.396
Male	7.11 ± 4.79	7.24 ± 2.50	–0.205	0.839

WBC: white blood cells; CRP: C-reactive protein

## Discussion

The field of use of cyanoacrylate (CA) in medical treatment has gradually increased since its discovery.^13^,^14^ It has been used in ophthalmological operations, cosmetic procedures, dental applications and acute bleeding, with the aim of stopping the bleeding and attaching the tissues. Also, endoscopic injection of CA has been widely and safely used in order to cease gastric variceal bleeding.^9^ Recently it has been administered via the endovenous route for the treatment of varicose veins and superficial venous insufficiency without the need for tumescent anaesthesia^11^ or thermal energy, with increasing evidence proving that it could be an appropriate agent for the treatment of peripheral varicose veins.^10^,^15^

The mechanism of effect of NBCA is simple; it stimulates polymerisation when it comes into contact with blood and plasma, hence causing an obstruction of the vein in which it is administered. This occurs in three steps: the initiation phase lasts approximately 10 seconds and the tensile force increases rapidly; the second phase lasts for approximately one minute and creates a steady tensile force; the last phase is completion of the polymerisation and a strong tensile force is obtained.^16^

Almeida et al. closed the truncal vein of pigs using CA and after a follow-up period of 60 days, found no thrombus obstructing the lumen of the vein on sonography or histology. Instead he observed a chronic foreign body reaction against NBCA.^15^ When the tissues were examined, they observed an inflammatory reaction and the formation of giant cell foreign bodies, followed by the development of intraluminal fibrosis.^15^

Endovenous NBCA application has been well tolerated in patients. The results of our administration are similar.

Inflammation is activated when an organism is triggered by stimulants. An acute inflammatory reaction is characterised by neutrophil predominance in the region of the event.^17^ Neutrophil and leukocyte counts in the blood are increased during acute inflammation. No statistically significant changes were detected between pre- and postoperative counts of either WBC or neutrophils in our study. Acute inflammation in the endovenous administration of NBCA was therefore most likely localised in the vein wall and surrounding tissues. There are reports in the literature demonstrating that NBCA causes a local inflammation,^10^,^12^ but there are no studies that have evaluated the systemic response.

The acute-phase response includes endocrinological, neurological and immunological events.^18^ Proteins, whose levels increase or decrease during this period, are called acute-phase proteins or acute-phase reactants.^19^ Change in the levels of acute-phase proteins demonstrate the presence and severity of inflammation.^20^

Cytokines are released as a response to stress by inflammatory cells such as neutrophils and macrophages. Interleukine-6, interleukine-1 and tumour necrosis factor-α induce CRP secretion from the hepatocytes.^21^ CRP has a pro- and antiinflammatory effect. Its pro-inflammatory effects result in the activation of the complement system and the induction of tissue factor and inflammatory cytokines from the monocytes, but its most important role is its anti-inflammatory effect.^22^

Erythrocyte sedimentation rate (ESR) is a frequently used test for the evaluation of acute-phase response.^23^ ESR increases from the start of the inflammation and resolution may take up to a month.^24^ In our study, no statistically significant change was seen in the CRP level and sedimentation rate between the preand post-procedure states of endovenous NBCA use. Changes in sedimentation rate from the pre- to the postoperative values by gender were statistically significant in the male patients (p < 0.05); however they were within the normal range, and postoperatively showed a decreasing trend. CRP levels were similar between the pre- and post-procedural states by gender and in the overall group of patients. Since there was no change demonstrated in the CRP level and sedimentation rate, and in the neutrophil and WBC counts, it can be concluded that NBCA did not cause an acute systemic inflammatory response.

Sensitivity has been detected in patients when NBCA was used to repair skin wounds, and also in individuals who were occupationally exposed to CA.^25^ In a study by Quinn et al., eosinophilic inflammation was detected at a rate of approximately 2% following NBCA use in the closure of intracranial arteriovenous malformations. The authors reported that no history of sensitivity against or exposure to CA was previously detected in those patients.^25^ When our patients was evaluated, no statistically significant changes in the pre- and postoperative eosinophil and basophil counts were found. From these results, we concluded that NBCA caused no allergic reaction in this patient group.

## Conclusion

The greatest advantage of the endovenous medical ablation method using NBCA is that tumescent anaesthesia and thermal energy are not necessary. In addition, since it causes no systemic allergic or acute inflammatory reaction, it appears safe to use. However, we suggest that evaluations should be performed in a larger group of patients to confirm the results.
